# Prevalence rates of borderline personality disorder symptoms: a study based on the Netherlands Mental Health Survey and Incidence Study-2

**DOI:** 10.1186/s12888-016-0939-x

**Published:** 2016-07-19

**Authors:** Margreet ten Have, Roel Verheul, Ad Kaasenbrood, Saskia van Dorsselaer, Marlous Tuithof, Marloes Kleinjan, Ron de Graaf

**Affiliations:** Netherlands Institute of Mental Health and Addiction, Da Costakade 45, 3521 VS Utrecht, The Netherlands; Centre of Psychotherapy De Viersprong; University of Amsterdam, Amsterdam, The Netherlands; Centre of knowledge for Personality Disorders, Utrecht, Pro Persona, Wolfheze, The Netherlands

**Keywords:** Borderline personality disorder symptoms, Population survey, Prevalence, Comorbidity, Disability, Health service use

## Abstract

**Background:**

Despite increasing knowledge of the prevalence of borderline personality disorder (BPD) in the general population, and rising awareness of mental disorders both as a categorical and a dimensional construct, research is still lacking on the prevalence of the number of BPD symptoms and their associated consequences, such as comorbidity, disability, and the use of mental health services) in the general population.

**Methods:**

Data were obtained from the second wave of the Netherlands Mental Health Survey and Incidence Study-2 (*N* = 5303), a nationally representative face-to-face survey of the general population. BPD symptoms were measured by means of questions from the International Personality Disorder Examination. Comorbidity of common mental disorders was assessed with the Composite International Diagnostic Interview version 3.0.

**Results:**

Of the total population studied, 69.9 % reported no BPD symptoms, while 25.2 % had 1–2 symptoms, 3.8 % had 3–4 symptoms, and 1.1 % had ≥ 5 BPD symptoms. The number of BPD symptoms reported was found to be positively associated with not living with a partner, having no paid job, and/or having a comorbid mood, anxiety or substance use disorder. Even after adjustment for sociodemographic characteristics and comorbidity, the number of BPD symptoms turned out to be uniquely associated with disability. It also showed a positive relationship with using services for dealing with mental health problems, although this relationship was strongly affected by the presence of comorbid disorders.

**Conclusions:**

Because even a relatively low number of BPD symptoms appears to be associated with psychiatric comorbidity and functional disability, not only full-blown BPD but also subthreshold levels of BPD symptoms need to be identified in clinical practice and research.

## Background

The epidemiology of borderline personality disorder (BPD) has been studied in various large adult population-based surveys, mainly in the United States. These studies have shown that the prevalence rates for BPD vary between 0.5 % [1] and 1.4 % [2–6] of the total population. Two studies, based on data from the National Epidemiologic Survey on Alcohol and Related Conditions, have found higher rates, of 2.7 % [7] and 5.9 % [8] respectively, depending on how strictly the diagnostic rules are applied. A prudent assumption seems to be that, generally speaking, the population prevalence rate of BPD is circa 1 % [9].

Regardless of differences in prevalence rates, there is consistent evidence for a high comorbidity of BPD with common mental disorders, such as mood, anxiety and substance use disorders (e.g. [3, 5, 7, 8]). Yet it remains unclear whether, after adjustment for comorbidity with common mental disorders, BPD in the general population is also linked to mental disability and the use of services for mental health problems. There is only little research available on this topic, and existing studies show mixed findings [5, 7, 8, 10, 11].

In recent years, the awareness has risen that mental disorders can be viewed as both as a categorical construct (i.e. the presence or absence of a certain disorder) and a dimensional construct (i.e. a severity dimension, ranging from normality with hardly any symptoms to a full-blown disorder, when someone displays a minimum number of symptoms and suffers from associated disability). Although personality pathology is probably best described by a single severity dimension [12, 13], we are not aware of any research having been conducted on the prevalence rates of various numbers of BPD symptoms (an indication of severity) in the general population, and the associated consequences of such symptoms.

This paper attempts to fill this research gap by analysing data from the second wave (*N* = 5303) of the Netherlands Mental Health Survey and Incidence Study-2 (NEMESIS-2), which is a nationally representative survey of the general adult population. Our main research questions are: (1) What is the prevalence of various numbers of BPD symptoms in the general population, and what are their associated sociodemographic correlates? (2) To what extent does the number of BPD symptoms people display co-occur with common mental disorders? (3) After adjustment for sociodemographic characteristics and comorbidity, to what extent is mental disability associated with the number of BPD symptoms? (4) Is health care mainly sought for BPD symptoms or for the associated disorders? To answer these questions, we defined four categories of number of BPD symptoms, namely, having 0, 1–2, 3–4 and ≥ 5 symptoms. This categorization reflects a certain severity dimension, ranging from having no BPD symptoms to potentially suffering from BPD. People with ≥ 5 BPD symptoms could be viewed as suffering from BPD, because they fulfil the required number of criteria for a BPD diagnosis. BPD symptoms were assessed by means of questions from the International Personality Disorder Examination.

## Methods

NEMESIS-2 is a psychiatric epidemiological cohort study of the Dutch general population aged 18 to 64 years. It is based on a multistage, stratified random sampling of households, with one respondent randomly selected in each household. Based on the most recent birthday at first contact within the household, an individual aged 18–64 years with sufficient fluency in the Dutch language was randomly selected. Addresses of institutions and thus institutionalized individuals (i.e. those living in hospices, prisons) were excluded. Those temporarily living in institutions, however, could be interviewed later during the fieldwork if they returned home.

In the first wave (T_0_), performed from November 2007 to July 2009, a total of 6646 persons were interviewed (response rate 65.1 %; average interview duration: 95 min). This sample was nationally representative, although younger subjects were somewhat underrepresented [14]. The interviews were laptop computer-assisted and almost all were held at the respondent’s home.

All T_0_ respondents were approached for follow-up, three years after T_0_ from November 2010 to June 2012. Of this group, 5303 persons could be interviewed again (response rate 80.4 %, with those deceased excluded; average interview duration: 84 min). Attrition rate was not significantly associated with all main categories and individual 12-month mental disorders at baseline, after controlling for sociodemographic characteristics [15]. The mean period between both interviews was 3 years and 7 days.

For this paper, data from the second wave were used (*N* = 5303), because data on BPD symptoms were then collected.

The study was approved by a medical ethics committee (the Medical Ethics Review Committee for Institutions on Mental Health Care, METIGG). After having been informed about the study aims, respondents provided written informed consent. A more comprehensive description of the design is provided in De Graaf et al. [14].

The face-to-face interviews were conducted by trained professional interviewers of the fieldwork agency GfK (Growth from Knowledge) Panel Services Benelux, with their team of five supervisors. Interviewers were selected on their experience with systematic face-to-face data collection, experience with sensitive topics and ability to achieve a good response in other studies. Fieldwork was monitored over the entire data collection period by the NEMESIS-investigators and the fieldwork agency (for more information on quality checks of the data, see [14]). At the second wave, the best interviewers of the first wave were selected to do this job. To increase the response rate, respondents were as much as possible re-interviewed by the same interviewer as at the first wave.

### Borderline personality disorder (BPD) symptoms

BPD symptoms were measured by means of eight questions from the International Personality Disorder Examination (IPDE) [16, 17]; the questions form part of the Composite International Diagnostic Interview (CIDI) 3.0 [5, 18]. This measure uses a true-false response format, with scores being assigned on the basis of the total sum of ‘true’ responses. A higher score on this measure is indicative of a greater number of BPD symptoms. In a clinical reappraisal interview, performed in a subsample of the National Comorbidity Survey Replication (NCS-R) in the United States, it was found that such interview questions allow for a valid assessment of BPD [5].

For the present study, we defined four categories of number of BPD symptoms, namely, having 0, 1–2, 3–4, and ≥ 5 symptoms. People with ≥ 5 BPD symptoms could be viewed as suffering from BPD, because they fulfil the required number of DSM-IV criteria (at least 5 of the 9) for a BPD diagnosis.

### Comorbidity of common mental disorders

DSM-IV diagnoses of common mental disorders were made using the CIDI 3.0 - a fully structured lay-administered diagnostic interview. This instrument was developed and adapted for use in the World Mental Health Survey Initiative [19]. The CIDI 3.0 version used in NEMESIS-2 was an improvement on the Dutch one used in this initiative.

The comorbid disorders considered in this paper include: mood disorders (i.e. major depression, dysthymia, and bipolar disorder), anxiety disorders (i.e. panic disorder, agoraphobia without panic disorder, social phobia, specific phobia, and generalized anxiety disorder) and substance use disorders (i.e. alcohol/drug abuse and dependence). Comorbidity of any disorder refers to any of the above-mentioned mental disorders.

Clinical calibration studies in various countries [20] found that the CIDI 3.0 assesses mood, anxiety and substance use disorders with generally good validity in comparison to blinded clinical reappraisal interviews.

For this paper, the occurrence of comorbid disorders between baseline and follow-up were used.

### Vulnerability to mental disorders

To gain some further insight into the association between number of BPD symptoms and comorbid disorders, we also investigated the association between BPD symptoms and vulnerability to mental disorders. As vulnerability indicators we used: childhood abuse (whether before age 16 years one had experienced emotional neglect, psychological abuse, or physical abuse on two or more occasions, or sexual abuse on one or more occasion; these questions based on self-report were used before in the studies NEMESIS-1 and NESDA (the Netherlands Study of Depression and Anxiety) (see e.g. [21–24]); to increase the likelihood of these experiences being reported, they were not mentioned as such, but were listed in a booklet and referred to by number) and lifetime mental health problems of parents (at least one biological parent ever having been treated by a psychiatrist, or hospitalized in a mental institution, or ever having exhibited one or more of the following problems: severe depression, delusions or hallucinations, severe anxiety or phobias, alcohol abuse, drug abuse, regular problems with the police, and suicidal behaviour).

### Mental disability

Current mental disability was assessed with three subscales of the Medical Outcomes Study Short Form Health Survey (MOS SF-36) [25]: social functioning, role emotional functioning and mental health in the past 4 weeks.

Social functioning involves problems in one’s normal social activities as a result of somatic or emotional problems; a 2-item, 6-point subscale of the MOS SF-36 (Cronbach’s alpha = 0.78). Role emotional functioning involves problems at work or in other daily activities as a result of emotional problems; a 3-item, 2-point subscale of the MOS SF-36 (Cronbach’s alpha = 0.88). Mental health was assessed with a frequently used screener for mood and anxiety disorders [26]; a 5-item, 6-point subscale of the MOS SF-36 (Cronbach’s alpha = 0.79).

### Service use for mental health problems

Service use refers to at least one contact made in the general medical care or mental health care sector for emotional or addiction problems between baseline and follow-up. General medical care includes general practitioners, company doctors, social work, home care or district nurses, physiotherapists or haptonomists, medical specialists or other professionals working within this care sector. Mental health care includes psychiatrists, psychologists, psychotherapists, part-time or full-time psychiatric treatment.

### Sociodemographic characteristics

The sociodemographic characteristics and potential confounders used were: age (in four age categories because age is not always linearly related to the outcome measure, in this case BPD symptoms), gender, education level, living situation (i.e. living with a partner or not), and job status (i.e. having a paid job or not).

### Analyses

All analyses were performed with STATA version 12.1, using weighted data to correct for differences in the response rates in several sociodemographic groups at both waves and differences in the probability of selection of respondents within households at baseline. Robust standard errors were calculated in order to obtain correct 95 % confidence intervals and p-values [27].

First, sociodemographic characteristics of the four categories of number of BPD symptoms were calculated using simple descriptive analyses (Table 1). In addition to the overall *p*-values between two categorical variables (e.g. between gender and number of BPD symptoms), we calculated *p*-values between a certain sociodemographic variable and all possible combinations of BPD symptom categories (i.e. for gender, we calculated differences between the category with 0 symptoms compared to the category with respectively 1–2 symptoms, 3–4 symptoms, and ≥ 5 symptoms, et cetera; in total 6 possible comparisons between the four BPD symptom severity categories).

**Table 1:** Sociodemographic characteristics of categories with number of borderline personality disorder (BPD) symptoms in the general population (*N* = 5303), in weighted column percentages

	Total	0 BPD symptoms	1–2 BPD symptoms	3–4 BPD symptoms	≥5 BPD symptoms	*P* value
n (%)	5303 (100)	3783 (69.9)	1276 (25.2)	186 (3.8)	58 (1.1)	
Sociodemographic characteristics						
Female gender	49.5	50.1	46.0	56.0	72.6	.000**c**,**e**
Age at interview						.023
21–37	32.0	30.2	36.3	37.9	31.1	
38–47	24.5	24.1	24.8	27.3	30.9	
48–57	23.3	24.0	21.5	21.7	26.3	
58–67	20.2	21.7	17.4	13.0	11.7	
Education						.000**a**,b
Lower secondary	29.5	26.9	35.1	36.9	43.4	
Higher secondary	41.6	42.3	39.4	44.4	40.6	
Higher professional/university	28.8	30.8	25.5	18.7	15.9	
Living without partner	29.9	26.7	34.2	51.0	62.1	.000**a**,**b**,**c**,**d**,e
No paid job	24.8	23.2	25.6	43.1	47.4	.000**b**,**c**,d,e

a: category 0 symptoms versus category 1–2 symptoms was significantly different (*P* < .01; in bold at *P* < .001)

b: category 0 symptoms versus category 3–4 symptoms was significantly different (*P* < .01; in bold at *P* < .001)

c: category 0 symptoms versus category ≥ 5 symptoms was significantly different (*P* < .01; in bold at *P* < .001)

d: category 1–2 symptoms versus category 3–4 symptoms was significantly different (*P* < .01; in bold at *P* < .001)

e: category 1–2 symptoms versus category ≥ 5 symptoms was significantly different (*P* < .01; in bold at *P* < .001)

f: category 3–4 symptoms versus category ≥ 5 symptoms was significantly different (*P* < .01; in bold at *P* < .001)

Second, multivariate logistic regression analyses were performed to examine to what extent number of BPD symptoms is associated with a variety of common mental disorders, adjusted for gender and age (Table 2). By changing the reference category in the logistic regression analyses we were able to calculate the differences between all possible combinations of number of BPD symptom categories on comorbid disorders. Results of multivariate logistic regression analyses are usually expressed in adjusted odds ratios, but could also be expressed in adjusted average predicted probabilities by using the margins command (http://www.ats.ucla.edu/stat/stata/dae/logit.htm). We opted for this last mode, because it enabled us to show the probabilities of mental disorders in all BPD symptom severity categories (including the reference category). To gain further insight into the consistency of the association between number of BPD symptoms and comorbid disorders, we also investigated the association between BPD symptoms and vulnerability to mental disorders in the same way as described above.

**Table 2:** Clinical characteristics of categories with number of borderline personality disorder (BPD) symptoms in the general population (*N* = 5303), in weighted average predicted probabilities adjusted for gender and age (%)

	Total	0 BPD symptoms	1–2 BPD symptoms	3–4 BPD symptoms	≥5 BPD symptoms	*P* for trend
	%	% [95 % CI]	% [95 % CI]	% [95 % CI]	% [95 % CI]	
3-year mental disorders						
Any mood disorder	8.4	3.76 [2.87,4.66]	14.46 [11.87,17.04]	36.18 [27.74,44.62]	58.00 [40.27,75.74]	.000**a**,**b**,**c**,**d**,**e**
Major depression	7.5	3.45 [2.63,4.28]	13.52 [11.03,16.01]	30.67 [23.21,38.12]	40.53 [24.86,56.20]	.000**a**,**b**,**c**,**d**,**e**
Dysthymia	0.7	0.03 [–0.03,0.09]	1.41 [0.32,2.49]	3.93 [–0.90,8.77]	5.41 [–1.55,12.38]	.000a,**b**,**c**
Bipolar disorder	0.8	0.31 [0.03,0.58]	0.80 [0.18,1.41]	4.53 [1.61,7.45]	15.74 [4.60,26.88]	.000**b**,**c**,d,**e**
Any anxiety disorder	7.9	4.87 [3.88,5.87]	11.95 [8.60,15.31]	25.01 [18.37,31.65]	40.25 [27.24,53.27]	.000**a**,**b**,**c**,**d**,**e**
Panic disorder	1.9	0.88 [0.41,1.36]	3.81 [1.89,5.73]	4.38 [2.42,6.34]	11.58 [2.12,21.04]	.000**a**,**b**,**c**
Agoraphobia	0.4	0.29 [–0.01,0.60]	0.33 [0.09,0.57]	2.86 [–0.04,5.76]	3.35 [–0.55,7.25]	.004b,c,d,**e**
Social phobia	2.5	1.15 [0.57,1.74]	3.82 [2.16,5.48]	11.95 [6.62,17.28]	19.94 [4.97,34.91]	.000a,**b**,**c**,d,**e**
Specific phobia	3.5	2.68 [1.87,3.49]	4.71 [2.87,6.55]	7.41 [3.86,10.96]	13.15 [4.49,21.80]	.000b,**c**
GAD	1.6	0.77 [0.43,1.10]	2.77 [1.36,4.19]	6.89 [3.19,10.60]	5.32 [–0.80,11.44]	.000a,**b**,c,d
Any substance use disorder	5.7	3.82 [2.88,4.76]	7.40 [5.49,9.31]	18.58 [11.80,25.36]	31.32 [17.46,45.17]	.000**a**,**b**,**c**,**d**,**e**
Alcohol abuse	3.6	3.05 [2.18,3.92]	4.73 [3.04,6.41]	4.32 [1.49,7.15]	3.47 [–3.20,10.14]	.030
Alcohol dependence	0.9	0.24 [0.08,0.39]	1.12 [0.56,1.69]	7.27 [2.79,11.75]	19.23 [2.23,36.23]	.000**a**,**b**,**c**,**d**,**e**
Drug abuse	0.9	0.60 [0.14,1.07]	1.03 [0.18,1.88]	2.57 [–0.10,5.23]	8.16 [–0.33,16.65]	.003**c**,e
Drug dependence	0.6	0.08 [–0.00,0.16]	0.74 [0.23,1.25]	5.72 [0.30,11.14]	8.44 [–0.32,17.19]	.000**a**,**b**,**c**,d,e
Any mental disorder	17.4	10.96 [9.59, 12.33]	25.97 [22.10, 29.84]	57.18 [48.11,66.26]	74.76 [62.99,86.53]	.000**a**,**b**,**c**,**d**,**e**
3-year suicidal behaviour	2.4	0.94 [0.48,1.40]	3.61 [2.13,5.08]	14.63 [8.03,21.23]	29.87 [16.37,43.38]	.000**a**,**b**,**c**,**d**,**e**
Vulnerability to mental disorders						
Childhood abuse	27.3	22.83 [21.28,24.38]	35.31 [31.76,38.85]	48.63 [40.20,57.06]	58.12 [42.01,74.23]	.000**a**,**b**,**c**,d,e
Lifetime mental health problems of parents	31.4	28.58 [26.32,30.83]	36.07 [32.36,39.78]	42.05 [34.32,49.77]	65.90 [54.73,77.06]	.000**a**,**b**,**c**,**e,**f

a: category 0 symptoms versus category 1–2 symptoms was significantly different (*P* < .01; in bold at *P* < .001)

b: category 0 symptoms versus category 3–4 symptoms was significantly different (*P* < .01; in bold at *P* < .001)

c: category 0 symptoms versus category ≥ 5 symptoms was significantly different (*P* < .01; in bold at *P* < .001)

d: category 1–2 symptoms versus category 3–4 symptoms was significantly different (*P* < .01; in bold at *P* < .001)

e: category 1–2 symptoms versus category ≥ 5 symptoms was significantly different (*P* < .01; in bold at *P* < .001)

f: category 3–4 symptoms versus category ≥ 5 symptoms was significantly different (*P* < .01; in bold at *P* < .001)

Third, multivariate linear and logistic regression analyses were used to examine to what extent number of BPD symptoms is associated with mental disability and service use for mental health problems (Table 3), adjusted for sociodemographic characteristics (model 1) and additionally for any comorbid mood, anxiety or substance use disorder (model 2). Again, by changing the reference group in the regression analyses we were able to calculate the differences between all possible combinations of number of BPD symptom categories with regard to mental disability and health service use. The results were expressed in adjusted averages and adjusted average predicted probabilities, respectively, by using the margins command, to be able to show the averages on mental disability and the probabilities of health service use in all BPD symptom severity categories (including the reference category).

**Table 3:** Current mental disability and service use for mental health problems of categories with number of borderline personality disorder (BPD) symptoms in the general population (*N* = 5303), in weighted adjusted averages (Mean) or adjusted average predicted probabilities (%)

	Total	0 BPD symptoms		1–2 BPD symptoms		3–4 BPD symptoms		≥5 BPD symptoms		*P* for trend	
		Model 1	Model 2	Model 1	Model 2	Model 1	Model 2	Model 1	Model 2	Model 1	Model 2
	Mean	Mean [95 % CI]	Mean [95 % CI]	Mean [95 % CI]	Mean [95 % CI]	Mean [95 % CI]	Mean [95 % CI]	Mean [95 % CI]	Mean [95 % CI]		
Mental disability											
Social functioning^a^	89.7	92.07 [91.32,92.82]	91.48 [90.73,92.23]	87.28 [85.85,88.71]	88.06 [86.66,89.46]	68.85 [63.58,74.12]	72.52 [67.75,77.28]	63.72 [54.98,72.47]	70.60 [62.33,78.86]	.000**a**,**b**,**c**,**d**,**e**	.000**a**,**b**,**c**,**d**,**e**
Role emotional functioning^a^	92.2	95.35 [94.42,96.28]	94.51 [93.53,95.49]	89.62 [87.73,91.51]	90.72 [88.91,92.53]	63.80 [55.32,72.27]	69.06 [60.98,77.13]	51.40 [36.80,66.01]	61.32 [46.42,76.21]	.000**a**,**b**,**c**,**d**,**e**	.000**a**,**b**,**c**,**d**,**e**
Mental health^a^	80.1	82.92 [82.39,83.44]	82.51 [81.96,83.05]	75.91 [74.79,77.03]	76.46 [75.38,77.53]	63.80 [60.69,66.90]	66.31 [63.24,69.38]	54.20 [48.93,59.47]	58.99 [53.21,64.76]	.000**a**,**b**,**c**,**d**,**e**,f	.000**a**,**b**,**c**,**d**,**e**
	%	% [95 % CI]	% [95 % CI]	% [95 % CI]	% [95 % CI]	% [95 % CI]	% [95 % CI]	% [95 % CI]	% [95 % CI]		
3-year service use for mental health problems											
General medical care	16.0	10.55 [9.10,11.99]	13.19 [11.61,14.77]	23.52 [20.16,26.87]	19.75 [17.22,22.28]	47.76 [39.11,56.41]	30.04 [21.57,38.51]	64.91 [49.00,80.83]	33.09 [16.88,49.30]	.000**a**,**b**,**c**,**d**,**e**	.000**a**,**b**,c
Mental health care	11.1	6.68 [5.58,7.79]	8.57 [7.26,9.88]	16.68 [12.99,20.38]	13.87 [10.97,16.78]	37.55 [29.14,45.96]	22.39 [15.64,29.14]	49.35 [34.59,64.12]	21.35 [10.20,32.51]	.000**a**,**b**,**c**,**d**,**e**	.000**a**,**b**,c

a: category 0 symptoms versus category 1–2 symptoms was significantly different (*P* < .01; in bold at *P* < .001)

b: category 0 symptoms versus category 3–4 symptoms was significantly different (*P* < .01; in bold at *P* < .001)

c: category 0 symptoms versus category ≥ 5 symptoms was significantly different (*P* < .01; in bold at *P* < .001)

d: category 1–2 symptoms versus category 3–4 symptoms was significantly different (*P* < .01; in bold at *P* < .001)

e: category 1–2 symptoms versus category ≥ 5 symptoms was significantly different (*P* < .01; in bold at *P* < .001)

f: category 3–4 symptoms versus category ≥ 5 symptoms was significantly different (*P* < .01; in bold at *P* < .001)

Model 1: adjusted for sociodemographic characteristics (gender, age, education level, living situation, paid job status)

Model 2: adjusted for sociodemographic characteristics (model 1) as well as for mental disorders (any mood, any anxiety, any substance use disorder)

^a^ This scale varies from 0 (low functioning/ill health) to 100 (high functioning/good health)

Two-tailed testing procedures were used with 0.05 alpha levels in the main analyses except the tests to calculate the differences between all possible combinations of number of BPD symptom categories on a certain variable, where alpha levels of 0.01 and 0.001 were used, because of the large number of analyses.

## Results

Of the population studied, 69.9 % reported having no BPD symptoms, while 25.2 % had 1–2 symptoms, 3.8 % had 3–4 symptoms, and 1.1 % had ≥ 5 BPD symptoms. The number of BPD symptoms turned out to be significantly related to gender, age, education level, living situation, and job status (Table 1).

With respect to gender, the category of people with ≥ 5 symptoms included a significantly higher proportion of females as compared to the categories of people with 0 and 1–2 symptoms. Age differences were less pronounced, although the category with 0 symptoms consisted of slightly older respondents. The latter category was further characterized by a significantly larger proportion of higher professionals or university educated adults, as compared to the categories that had 1–2 and 3–4 symptoms. The differences between all possible combinations of number of BPD symptom categories were most pronounced with respect to the proportion of respondents living without a partner and those without a paid job. Generally speaking, the more BPD symptoms a respondent had, the more likely it was he or she was not living with a partner and had no paid job.

After adjustment for gender and age, respondents with a higher number of BPD symptoms turned out to be significantly more likely to suffer from various common mental disorders (Table 2). It was also found that, for the three main groups of mental disorders, as well as for any comorbid disorder, the differences between all possible combinations of number of BPD symptom categories reflected the anticipated trend and were highly significant, except for the difference between the two categories with the larger number of BPD symptoms. Although the category with ≥5 symptoms had higher average predicted probabilities of the main groups of mental disorders and any mental disorder compared to the category with 3–4 symptoms, these differences were not significant at *p* < .01, probably because of the small number of respondents in these categories (58 and 186, respectively). A similar picture was found for suicidal behaviour and childhood abuse: respondents with a higher number of BPD symptoms were more likely to report such experiences; the differences between the category with 3–4 and the category with ≥ 5 symptoms were not significant at *p* < .01. However, these last two categories did differ with respect to the proportion of parents with mental health problems. Whereas a minority of those with 3–4 symptoms had a parent with a psychiatric history, the reverse was true for respondents with ≥ 5 symptoms.

A comparison of the mental disability scores of those with different numbers of BPD symptoms showed that a higher number of BPD symptoms was positively associated with a higher likelihood of mental disability, even after adjustment for sociodemographic characteristics and any comorbid mood, anxiety or substance use disorder (Table 3). This means that the severity of BPD symptoms is uniquely related to higher mental disability scores. Again, we found that the differences between all possible combinations of number of BPD symptom categories were incremental and highly significant, except for the two categories with the largest number of symptoms. As this implies, a larger number of BPD symptoms is associated with higher mental disability scores. Although the category with ≥ 5 symptoms had lower averages for social and role emotional functioning and mental health compared to the category with 3–4 symptoms, these differences were not significant at *p* < .01; again, this is probably due to the small number of respondents in these categories.

A different picture emerges when it comes to the use of health services by respondents with a relatively high number of BPD symptoms. After adjustment for sociodemographic characteristics, respondents with a larger number of BPD symptoms turned out to be significantly more likely to use services for their mental health problems. However, these probabilities were substantially lower after adjustment for additional comorbid mood, anxiety or substance use disorders; the number of BPD symptoms was still uniquely related to the use of health services, but respondents appeared to seek care mainly for comorbid mental disorders. As to the differences between all possible combinations of number of BPD symptom categories, significant differences were only found between respondents with 0 symptoms on the one hand and respondents with 1–2, 3–4 and ≥ 5 symptoms on the other. This means that, although the number of BPD symptoms is indeed significantly related to a more frequent use of health care services, the main distinguishing factor is whether or not someone has any BPD symptoms at all.

## Discussion

### Key findings

To the best of our knowledge, this is the first study which relates the number of BPD symptoms in the general population to comorbidity of common mental disorders and mental disability. Previous population studies have confined themselves mostly to assessing the prevalence rate of BPD diagnosis and its associated correlates and consequences. The present study builds on existing knowledge by showing that, in a representative population sample of adults, even low numbers of BPD symptoms are associated with psychiatric comorbidity and functional disability. Furthermore, it demonstrates that the number of BPD symptoms is associated with more frequent use of services for mental health problems, but that such care is mainly sought for comorbid mental disorders.

### Strengths and limitations

For this paper, data were used from NEMESIS-2. Although for most parameters NEMESIS-2 is representative of the Dutch population, people with an insufficient mastery of Dutch, those with no permanent residential address and the institutionalized are underrepresented in this sample. Hence, our findings cannot be generalized to these groups.

BPD symptoms were measured with eight questions from the International Personality Disorder Examination (IPDE). Despite indications of the validity of these IPDE questions for assessing BPD diagnoses [5], its properties make it unsuitable for use in clinical practice. However, the questionnaire can be used in epidemiological studies where the focus is on determining prevalence rates and associated correlates among groups of individuals. The present study yields clear indications of its validity, showing that respondents with a higher number of BPD symptoms are consistently more likely to report suicidal behaviour, all main groups of mental disorders, a vulnerability to these disorders, and a higher prevalence of mental disability. In order to establish the extent to which the present findings can be replicated, future population studies need to go beyond the exclusive use of screening questions and employ clinical interviews to assess full borderline personality disorder, as well as clinical reappraisal interviews to validate existing screeners for borderline personality disorder.

Just as in the NCS-R [28], in our study one criterion for BPD (namely, recurrent suicidal behaviour, gestures or threats, or self-mutilating behaviour) was not assessed in the IPDE screening questionnaire. However, in the suicidality module of the CIDI 3.0, respondents were asked about whether or not they had ever had experienced suicidal ideation, or made suicide plans and/or attempts. As expected, the more BPD symptoms respondents had, the more often they reported having experienced suicidal behaviour in the preceding years; this confirms the validity of the IPDE questionnaire.

Underreporting and recall problems might conceivably have compromised the assessment of respondents of any BPD symptoms they might have and might have had, especially in those cases where such symptoms occurred a long time ago [29]. The IPDE questions refer to rather stable personality characteristics (i.e. ‘What you are like most of the time?’ ‘What has been typical of you throughout your life?’). Mental disorders were assessed over a 3-year period, which could increase the likelihood of recall bias. Besides, it is conceivable that people with BPD symptoms perceive their mental health in a more negative way. We tried to minimize this type of bias by using a sound diagnostic instrument for the assessment of mental disorders. All in all, it is difficult to estimate the exact extent to which underreporting, recall or report problems may have influenced our findings.

In NEMESIS-2, the most common mental disorders were assessed. However, not all cluster B personality disorders were recorded in the dataset: the same goes for cluster A and cluster C personality disorders. This means that impairment associated with these disorders and the use of health care services could not be studied. Previous research has shown, however, that it is comorbid common mental disorders and not comorbid personality disorders that ultimately determine the social functioning of people with BPD in the general population; the same goes for seeking treatment [10]. As this suggests, the associations found were hardly affected by our inability to adjust for other personality disorders.

Our study relates the number of BPD symptoms to comorbidity of common mental disorders and mental disability in the general population. Most population studies conducted thus far have mainly confined themselves to assessing the prevalence rate of BPD diagnosis and its associated correlates and consequences. Huang et al. (2009), for example, show that the prevalence rates of personality disorders (PD) differ between countries, although the sociodemographic correlates of PD and comorbidity with PD of different countries show great similarity [18]. This implies that our findings on the prevalence rates of BPD symptoms can perhaps not be generalized to other countries. The associations between the number of BPD symptoms and sociodemographic characteristics, comorbidity of common mental disorders and/or mental disability, on the contrary, could be generalized to other countries.

### Discussion of research findings

In our study, 1.1 % of the population studied reported ≥ 5 BPD symptoms. These individuals could be viewed as having BPD, because they fulfil the required number of DSM-IV criteria for a BPD diagnosis. This finding concurs with the assumption made by Lenzenweger et al. [9] that the average population prevalence of BPD is circa 1 %.

The finding that a greater number of BPD symptoms is associated with less stable social situations (i.e. not living with a partner, or having no paid job) confirms earlier research (e.g. [7, 10, 28]) and is partly inherent in the definition of BPD; after all, the main characteristic of BPD is that someone shows a pervasive pattern of instability in interpersonal relationships, self-image, and emotions.

One of the findings of our study was that the category of people with ≥ 5 symptoms consisted of a significantly higher proportion of females as compared to the categories of people with 0 or 1–2 symptoms. This contrasts with previous population studies, which have shown no gender differences in the prevalence rate of BPD (e.g. [4, 5, 10]); yet it confirms the suggestion made in the DSM-5 that BPD is more common among women [30] and also substantiates the findings of clinical studies, which have demonstrated that more women than men suffer from BPD. However, the gender difference in clinical studies may result from selection bias (i.e. women seeking health care more often than men do) [31]. As a second point, additional analyses based on data from NEMESIS-2 have revealed that females and males differ significantly in terms of the type of BPD symptoms from which they suffer. Females more often report so-called ‘disturbed relatedness’ symptoms, in particular: ‘I often feel “empty” inside’ (7.9 % versus 3.6 %), and: ‘When I’m under stress, things around me don’t seem real’ (8.4 % versus 3.7 %); males, on the other hand, more often report: ‘I go to extremes to try to keep people from leaving me’ (10.4 % versus 7.0 %), which is a symptom of the affective dysregulation dimension [32]. It is unclear whether the gender differences in the number of BPD symptoms found in the present study reflect any real differences, or whether women are more likely to report certain BPD symptoms.

Similar to previous findings (e.g. [7, 8, 10, 18]), in our study the number of BPD symptoms turned out to be negatively related to age and education level. As regards age, this could result from the phenomenon of particular (i.e. impulsive) symptoms declining as people grow older [2, 9]; the education effect could be a consequence of the disorder itself. After all, BPD is characterized by impulsive behaviour and an unstable pattern of interacting with others, which could impede educational achievements.

After adjustment for gender and age, respondents with a higher number of BPD symptoms were found to be significantly more likely to suffer from various mental disorders, including mood, anxiety and substance use disorders. This is consistent with previous research, which has shown that BPD is strongly comorbid with a variety of mental disorders (e.g. [3, 5, 7, 8]) and is closely associated with both the distress sub-factor of the latent internalizing dimension and the latent externalizing dimension of common mental disorders [33]. These findings lead us to question the extent to which common mental disorders and personality disorders should be viewed as distinct, and also the extent to which BPD can be distinguished clearly from normal variation [12]. Future research might shed more light on these topics, which could have useful implications for both clinical practice and the mental health care structure as a whole. With the elimination of the multi-axial system in the DSM-5, some artificial distinctions between personality disorders and other common mental disorders (e.g. mood, anxiety and substance use disorders) have already disappeared.

The finding that, after adjustment for sociodemographic characteristics and comorbid mental disorders, higher numbers of BPD symptoms are uniquely related to higher mental disability scores is in line with previous research, which has found an independent contribution of BPD to mental disability [7, 8] and an independent contribution of cluster B personality disorders to the impairment of role functioning and social interaction [18]. However, our findings contradict another study, which has found largely reduced effects of BPD on impaired functioning after adjustment for comorbid mental disorders [5].

As our study shows, people with a higher number of BPD symptoms are significantly more likely to use certain services to deal with their mental health problems; after adjustment for comorbid mental disorders, however, these differences largely disappear. This corroborates previous research, in which higher rates of use of mental health services were found for people with BPD as compared to people with any mental disorder [11], and that people with any personality disorder often seek treatment for comorbid common mental disorders [18]. However, it contradicts other research which shows that, after adjustment for demography and comorbid common mental and personality disorders, people with BPD are not more likely to seek help from mental health professionals [5, 10].

All in all, our findings suggest that people with BPD seek help mainly for common mental disorders, even though most of their impairments do not result directly from these comorbid mental disorders. Thus, it seems that help-seeking as well as referral to specialized mental health care by general practitioners is often based on symptoms rather than traits, probably due to the perception, among both patients and professionals, of there being more effective treatment options available for common mental disorders than for BPD.

## Conclusions

Following Tyrer et al. [12], we recommend that not just full-blown BPD but also subthreshold levels of BPD symptoms be identified more frequently in clinical practice and research than is presently the case, as even low numbers of BPD symptoms were associated with psychiatric comorbidity and functional disability.

## Abbreviations

BPD, borderline personality disorder; CIDI, Composite International Diagnostic Interview; DSM, diagnostic and statistical manual of mental disorders; GfK, growth from knowledge; IPDE, International Personality Disorder Examination; METIGG, Medical Ethics Review Committee for Institutions on Mental Health Care; MOS SF-36, Medical Outcomes Study Short Form Health Survey; NCS-R, National Comorbidity Survey Replication; NEMESIS-2, Netherlands Mental Health Survey and Incidence Study-2; NESDA, Netherlands study of depression and anxiety; PD, personality disorders; STATA, a general-purpose statistical software package created in 1985 by StataCorp; T_0_, first wave
